# Graded expectations in visually situated comprehension: Costs and benefits as indexed by the N400

**DOI:** 10.3758/s13423-020-01827-3

**Published:** 2020-12-02

**Authors:** Maria Staudte, Christine Ankener, Heiner Drenhaus, Matthew W. Crocker

**Affiliations:** Department of Language Science and Technology, Saarland University Saarbrücken, Saarbrücken, Germany

**Keywords:** Word expectancy, Surprisal, Prediction, Processing effort, Visual world, Situated language processing

## Abstract

Recently, Ankener et al. (*Frontiers in Psychology, 9*, 2387, 2018) presented a visual world study which combined both attention and pupillary measures to demonstrate that anticipating a target results in lower effort to integrate that target (noun). However, they found no indication that the anticipatory processes themselves, i.e., the reduction of uncertainty about upcoming referents, results in processing effort (cf. Linzen and Jaeger, *Cognitive Science, 40*(6), 1382–1411, 2016). In contrast, Maess et al. (*Frontiers in Human Neuroscience, 10*, 1–11, 2016) found that more constraining verbs elicited a higher N400 amplitude than unconstraining verbs. The aim of the present study was therefore twofold: Firstly, we examined whether the graded ICA effect, which was previously found on the noun as a result of a likelihood manipulation, replicates in ERP measures. Secondly, we set out to investigate whether the processes leading to the generation of expectations (derived during verb and scene processing) induce an N400 modulation. Our results confirm that visual context is combined with the verb’s meaning to establish expectations about upcoming nouns and that these expectations affect the retrieval of the upcoming noun (modulated N400 on the noun). Importantly, however, we find no evidence for different costs in generating more or less specific expectations for upcoming nouns. Thus, the benefits of generating expectations are not associated with any costs in situated language comprehension.

## Introduction

Current themes of human language processing emphasize the role of predictive mechanisms, in which expectations about the upcoming words are determined by the linguistic context. Evidence comes from the findings that word expectancy—or its surprisal—correlate with processing effort (e.g., Kutas & Hillyard, 1980; Federmeier et al.,, 2007; Van Berkum et al.,, 2007; Demberg & Keller, 2008; Smith & Levy, 2013). Moreover, the visual world paradigm has provided evidence in support for both predictive language processing (Altmann & Kamide, 1999) as well as the influence of the *visual scene* on language understanding (Knoeferle et al., 2005). However, the attentional measures used in this paradigm provide no direct evidence regarding processing effort (neither for the formation of expectations nor for the processing of more or less expected words). The present study aims to address this gap by examining how word expectancy, as determined jointly by both *visual and linguistic* content, affects processing effort, both when expectations are *generated* and on the more or less expected word itself.

Word expectancy, or predictability, can be derived using the information-theoretic measure of surprisal, which denotes the negative logarithm of the likelihood of that word to come up in a given context (Shannon, 1949; Hale, 2001). It has been common recently to estimate word expectancy using precisely this notion, typically by sampling from human judgments, as in e.g., Cloze tasks, or using language models trained on large corpora (e.g., Roark et al.,, 2009; Frank, 2013b). Word expectancy has also been correlated with processing effort, in that less expected words take more effort to process (e.g., Kutas & Hillyard, 1980; Hale, 2001). The cognitive processing effort, in turn, has typically been assessed through measuring reading times or event-related potentials (ERPs) during word-by-word comprehension (e.g., Dambacher & Kliegl, 2007; Smith & Levy, 2013). In particular, ERP components such as the N400 have been shown, among other things, to correlate with surprisal estimates from language-models for a given word (Frank et al., 2015). Here, we specifically adopt the view that the more expected a word is, the easier it is to retrieve and integrate with the context, as suggested by Brouwer et al., (2012). Under this account, the N400 indexes facilitated or inhibited retrieval as a result of stronger or weaker expectations. Nevertheless, the correlation between expectancy and any index of processing effort is still based on a rather indirect combination of two separate measures (often from different people) on the linguistic materials: firstly the offline collected values for the conditional likelihood of a particular word, and secondly the processing time or amplitude for that word (e.g., Wlotko and Federmeier, 2012).

The visual world paradigm (VWP) helps examine more directly, and online, *what* expectations are formed during language comprehension: Anticipatory eye-movements to displayed objects or actions provide insight into which event participants the listener expects to hear next (Altmann & Kamide, 1999). Such eye movements provide a direct and online measure of the expectations that listeners generate for upcoming words and thus add another valuable and online index of predictability. At the same time, expectations for the next word(s) are not only made explicit and observable through the attention in the visual scene, they may also be influenced by the scene itself through depicted events and thematic roles (e.g., Knoeferle et al.,, 2005). What remains unclear is a) how the attentional measures used in this paradigm can be linked to processing effort, and b) whether the visual context helps to reduce uncertainty about upcoming words, similar to linguistic context, such that it also modulates processing effort – as would be predicted by the entropy reduction hypothesis (Frank, 2013a; Linzen & Jaeger, 2016).

Ankener et al., (2018) recently used a combination of the VWP and a measure of effort, enabling them to examine precisely that: they *simultaneously* investigated the influence of visual context on expectations and the associated processing effort. Specifically, they deployed a pupillary measure, namely the Index of Cognitive Activity (ICA; see Marshall, 2000), as an index of cognitive processing effort to observe the direct effect of visual context on word expectations and processing load (Demberg and Sayeed, 2016; Sekicki & Staudte, 2018; Tourtouri et al., 2019). They presented German sentences such as “Der Mann verschüttet gleich das Wasser.” (English word-by-word translation: The man spills now the water) simultaneously with a visual display that featured a varying number of objects matching the verbal constraints: either one, three, four, or none of the displayed objects were actually *spillable*. Thus, there was more or less temporary competition after the verb for upcoming object nouns based *solely* on the visual scene as the utterance did not vary within an item. Assuming that the probability for an upcoming verb argument is distributed among all plausible (visible) referents, a larger number of such referents would result in a lower likelihood for any individual one (lower surprisal in this specific *situation*), which in turn would increase processing effort on the given object noun. Depending on the number of matching objects, it was therefore hypothesized that the particular target noun (“water”) would become more or less predictable—and therefore would require more or less processing effort when it was actually mentioned. Thus, the manipulation of *number* of potential referents served as a means to modulate the likelihood for a given object noun to come up.

The results of the Ankener study replicate and extend findings by Altmann and Kamide (1999), in that they show that verbal constraints can drive anticipatory eye movements towards (all) matching objects in anticipation of the upcoming noun, even when more than one competitor is shown. Crucially, the authors found that the same object noun in the same linguistic context was more difficult to process (higher ICA values) when more depicted objects matched the verb constraint, making the actual target word less predictable. These results suggest that visually determined expectations for a spoken target word determine its *situated surprisal* and that this elicits processing effort for that word accordingly.

Interestingly, this study did not find any modulation of processing effort on the verb, where expectations are generated and, importantly, distinct between the conditions. While the anticipatory eye movements clearly index distinct expectations in this time window, the ICA values suggest that processing the verb (“spill”) required the exact same effort in all visual contexts. This result appears to be in conflict with the result presented by Maess et al., (2016), who compared sentences such as “Er dirigiert das Orchester” and “Er leitet das Orchester” (English translation: He conducts/leads the orchestra) in an MEG study. They found that “orchestra” was easier to process, as reflected by a reduced N400, after the more constraining verb “conducts” compared to the less constraining verb “leads”.^1^ At the same time, they also found that the constraining verb “conducts” elicited itself an increased N400 compared to the less constraining verb. This pattern was very tentatively interpreted as a trade-off between constraint and expectancy:

*“The predictive-verb N400 and the less predicted-noun N400 was inversely correlated which demonstrates a direct trade-off in terms of neural expenditure between the predictive and the predicted stage (...)”* (p.8, Maess et al.,, 2016).

There are several possible explanations for the diverging results in these two studies. But most notably, the Maess study found differential effects on *different* verbs (inherently more or less constraining), whereas the Ankener study found similar values for the *same* verb, when it was more or less constraining depending on the *visual* context. The two studies further employed different measures and the null effect in the pupillary measure ICA may be due to an insensitivity of the measure towards constraint effects. Generally, the ICA remains poorly understood in psycholinguistic paradigms, and it is unclear still precisely what aspects of processing effort it indexes.

The aim of the present study was therefore twofold: Firstly, we examined whether the graded ICA effect, which was previously found on the noun as a result of a likelihood manipulation, replicates in ERP measures. Secondly, we set out to investigate whether the processes leading to the generation of expectations (derived during verb and scene processing) induce an N400 modulation. If indeed a complementary N400 effect on the verb, compared to the noun (as in Maess et al.,, 2016), was found, this would support a “trade-off” theory suggesting that the benefit of specific expectations for noun processing comes along with additional effort during verb processing when uncertainty is reduced. This would also indicate that the ICA is sensitive to processing effort only when caused from selected mechanisms or sources. Alternatively, if no modulation on the verb was found, this would support the previous ICA results and suggest that using visual context to generate more or less specific expectations about upcoming content is not costly.

The current study uses the same design, stimuli (plus additional ones in order to achieve necessary power) and task as Ankener et al., (2018) and ERPs as the dependent measure in order to link expectation (generation) to effort of processing (see e.g., Kutas and Hillyard, 1980, Frank et al., 2015).

## Method

**Fig. 1:**
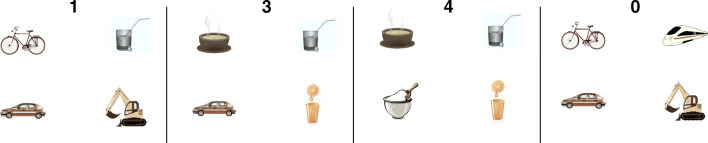
Sample conditions. Different visual contexts for the sentence *The man spills on Saturday the water in the kitchen*

We manipulated *only* the visual context, keeping the linguistic stimulus constant: A sentence containing a constraining verb (like *spill*) and an object noun of high thematic fit (*water*). Each sentence was presented along with four different visual contexts where the number of displayed objects matching the verb constraint was manipulated (e.g., **0**, **1**, **3**, or **4** “spillable” objects; see Fig. 1). Thus, linguistic surprisal was kept constant across conditions, while the same verb reduced visual uncertainty to different degrees, resulting in varying levels of expectancy for the target noun.

### Materials

We extended the stimuli set of Ankener et al., (2018) to increase power. A total of 96 plausible linguistic stimuli were combined with the visual displays in such a way that all four conditions of each display shared one sentence.

The visual scenes varied in the number of instantiations of a category that the verb selected, i.e., the potential referents. That is, either none, one, three, or all four of the pieces of clip art shown in a scene could be target referents matching the verb. All four clip art images in a display were pretested^2^ and arranged quadrangular around the center of the screen.

**Fig. 2:**
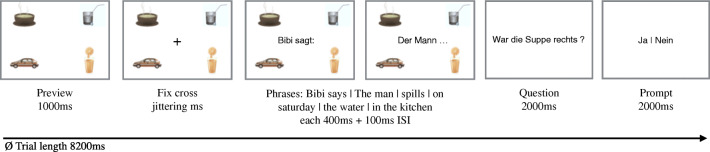
A trial time line example. The example scene shows three plausible target referents

An equal amount of fillers was added to introduce variation in terms of the number and type of matching and non-matching clip arts. All conditions of an item were distributed across four lists and randomized using the Latin square. In contrast to the Ankener study, all sentences in this experiment were presented in *written* segments (as in Fig. 2) in the center of the screen.

### Participants

Originally, 36 right-handed native speakers of German were recruited, but due to more than 20% eye artifacts, eight participants were removed from the analysis. The final 28 participants had a mean age of 24.7 years (range: [19, 34]; SD: 3.16; female: 22). All participants gave informed consent and were monetarily reimbursed (10 Euro/h).

### Procedure

Visual displays were presented, using E-Prime, with a 1000-ms preview time (see also Fig. 2), in which participants were allowed to move their eyes in order to identify and inspect the clip art items. Participants were seated in front of a 19” Dell 1908FP TFT UltraSharp monitor (resolution of 1280x1024 with a refresh rate of 75 Hz). The distance between the participant and the screen was always 103 cm in order to keep all of the objects in a 5^∘^ visual angle from the center of the screen. As soon as a fixation cross appeared for a variable duration in the middle of the display, participants were asked to keep their eyes focused on the cross. Words were then presented for 400 ms, with a 100-ms inter-stimulus interval. The visual displays stayed on the screen for the entire trial time. Subsequent to the sentences, the visual displays disappeared and a question appeared on the screen concerning either the visual (e.g., *Was the milk on the right?*), or the linguistic content (e.g., *Did the man spill the milk?*). Subjects were asked to answer using a button press with a new assignment of correctness to buttons in each trial.

### Analysis

The EEG was recorded by means of 24 Ag/AgCl scalp electrodes (actiCAP, BrainProducts) amplified with a BrainAmp (BrainVision) amplifier. Electrodes were placed by the 10–20 system (Sharbrough et al., 1995). Impedances were kept below 5 kOhm. The signal was digitized at a sampling rate of 500 Hz, referenced online to the reference electrode (FCz) and offline to the average of both mastoid electrodes. The AFz electrode was used as the ground electrode. The horizontal electrooculogram (EOG) was monitored with two electrodes placed at the outer canthus of each eye and the vertical EOG with two electrodes above (supraorbital) and below (infraorbital) the left eye. The EEG data were band pass filtered offline with 0.01–40 Hz (Luck, 2014). Single-participant averages were computed in a 800-ms window per condition relative to the onset of the critical item and aligned to a 200-ms pre-stimulus baseline and semi-automatically screened off-line for electrode drifts, amplifier blocking, eye movements, muscle artifacts (on average < 8%). Only artifact-free ERP averages from the 28 remaining participants time locked to the onset of the critical words were entered into the analysis.

All analyses were conducted using the *ez* package for R, to perform repeated measures analysis of variance (ANOVA) with Greenhouse–Geisser corrected *p* values. We analyzed a typical N400 time window between 300 and 500 ms after onset of the verb and noun. Main effects were assessed by running omnibus ANOVAs with electrode site (frontal/central/parietal ROIs) and experimental condition (number of competitors matching the verb) as within factors.

## Results

**Fig. 3:**
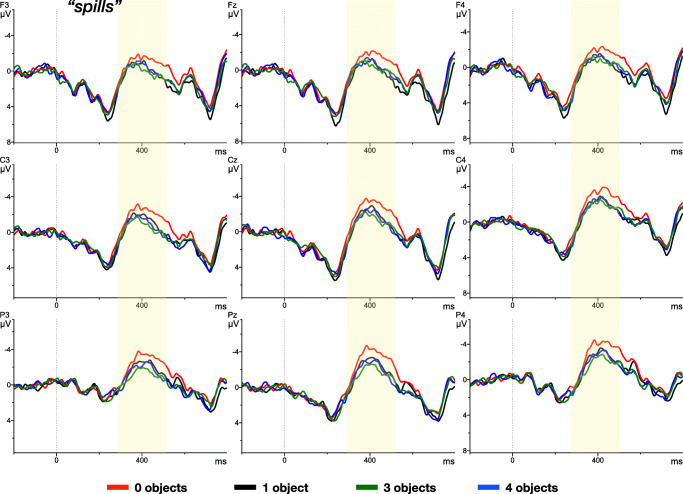
ERP time-locked to the onset of the VERB, e.g. “spills” (*dotted line*) and separated by the experimental conditions: one, three, four, no ’spillable’ referents in the scene. The data shows a subset of nine electrodes (unfiltered) for presentation purposes

In reporting the results, we first consider the verb region before turning to the object noun. The mean amplitude across the verb window for the individual conditions is plotted in Fig. 3. Visual inspection of this data suggests that only the mismatch condition **0** elicited an increased negativity at 400 ms after verb onset.

**Fig. 4:**
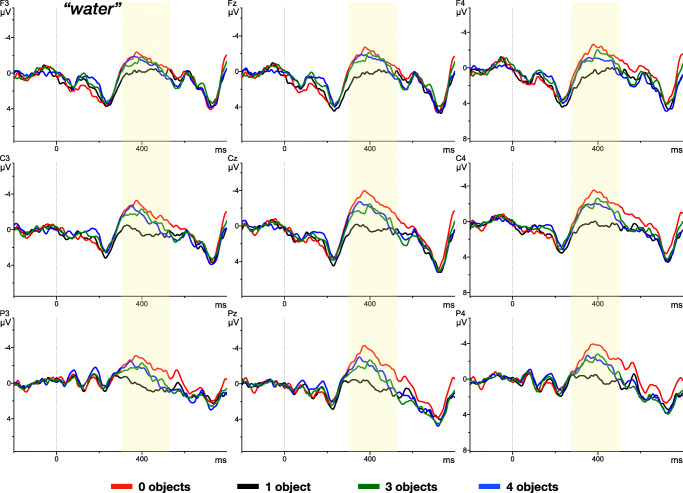
ERP time-locked to the onset of the NOUN, e.g., “water” (*dotted line*) and separated by the experimental conditions: one (= only glass of water), three (two additional), four (three additional), no ’spillable’ referents in the scene. The data shows a subset of nine electrodes (unfiltered) for presentation purposes.

The noun region is plotted in Fig. 4. In this time window, the plots suggest that all four conditions elicited a modulated ERP response to the more or less predictable target word. That is, the N400, peaking at 400 ms after onset of the critical word, might differ in amplitude between conditions.

An ANOVA assessed the statistical significance of these planned contrasts, revealing a main effect for condition (*F*(3.81) = 5.57, *p* < 0.05, *η*^2^ = 0.03) on the verb. Follow-up pairwise comparisons show that a significantly larger negativity was elicited by condition **0** (-1.23μV) compared to the baseline condition **1** (-0.73μV) (*F*(1.27) = 5.54, *p* < 0.05, *η*^2^ = 0.06). Negativity was widespread across frontal, central, and parietal regions, while being largest in the latter. However, conditions **3** (-0.77μV) and **4** (-0.45μV) did not yield significant differences in the N400 component, compared to **1** (Table 1).

**Table 1:** N400 amplitude differences on verb and noun region, *ezANOVA (dv = N400 value in each time window, wid = Subject, within = Targets, region)*

time window:	Factor	F-value	*eta*^2^	p
		(DFn, DFd)		(GG corrected for overall)
Verb				
Overall	Targets	5.57 (3,81)	.03	<.05
Follow-up	One vs. Zero poss. Targets	8.54 (1,27)	.03	<.05
Follow-up	One vs. Three poss. Targets	.001 (1,27)	.00	>.05
Follow-up	One vs. Four poss. Targets	1.48 (1,27)	.01	>.05
Noun				
Overall	Targets	7.41 (3,81)	.12	<.05
Follow-up	One vs. Zero poss. Targets	19.47 (1,27)	.21	<.05
Follow-up	One vs. Three poss. Targets	9.17 (1,27)	.10	<.05
Follow-up	One vs. Four poss. Targets	6.60 (1,27)	.08	<.05

In the noun window, we also found a main effect of condition (*F*(3.81) = 7.41, *p* < 0.05, *η*^2^ = 0.12). Specifically, condition **1**, in which the noun was most predictable, resulted in the lowest N400 amplitude (0.05μV). Conditions **3** (-0.73μV) and **4** (-0.68μV), where the target noun could be expected with 33% and 25% certainty, resulted in a significantly higher amplitude (three: *F*(1.27) = 9.17, *p* < 0.05, *η*^2^ = 0.09, four: *F*(1.27) = 6.60, *p* < 0.05, *η*^2^ = 0.07). Condition **0** (-1.23μV), where none of the clip art items in the visual display could be used to predict the target noun, yielded the highest difference in the N400 amplitude, compared to **1** (*F*(1.27) = 19.47, *p* < 0.05, *η*^2^ = 0.21).

## Discussion

Firstly, this experiment was designed to investigate whether the N400, which is known to index retrieval effort related to linguistic expectancy and surprisal, is also sensitive to target word expectancy and surprisal when the corresponding word expectations are modulated *only* through manipulations of the visual context. Secondly, the study aimed to examine the hypothesis that the processes leading to the generation of such expectations are effortful by themselves. This idea is supported by findings from Maess et al., (2016) and also the entropy reduction theory (Hale, 2016; Linzen and Jaeger, 2016). Ankener et al., (2018), however, found no evidence for this view using behavioral measures. We therefore re-examined this hypothesis using ERPs.

Results from the present experiment indeed revealed a globally distributed ERP response in reaction to the *same* noun, presented in different visual contexts. In particular, we observed a reduced N400 on the noun “water” when it had fewer competitors among the co-present visual objects and, thus, was most predictable. Conditions **3** and **4** did, however, not differ from each other. The similarity between these two conditions may be due to the inhibition of eye-movements in the EEG experiment, making a discrimination between 3 and 4 suitable objects more difficult, or to the low numeric difference in competitors for the two conditions.

Moreover, our results replicate and extend the findings using the pupillary measure in Ankener et al., (2018), showing no impact of constraint/uncertainty reduction on processing effort. There was, however, a significant mismatch effect in condition **0**, where nothing *spillable* was depicted and, thus, none of the objects matched the verbal constraints (see also Tourtouri et al., (2015) for a similar mismatch effect). The mismatch result in combination with the absence of an effect between the **1** and **3** & **4** conditions on the verb suggests that listeners carefully evaluate the linguistic stimulus against the visual context – but that there is no graded N400 during the verb for the three matching conditions. This suggests that listeners do not incur any cost associated with generating expectations. In addition, the increased N400 in the **0**-condition could reflect an inhibition of retrieving the verb, compared to the other conditions, where suitable objects could prime the action verb and therefore facilitate retrieval.

This result seems to be in conflict with the result found by Maess et al., (2016), but only at first sight. We argue that a crucial difference between the studies lies in the visual context. The advantage of the VWP, namely the opportunity of making expectations explicit through anticipatory eye movements, could also be viewed as a disadvantage: it makes processing mechanisms more complex as uncertainty and expectations may now be distributed between internal representations (and working memory) and the external representations on screen (Spivey et al., 2004). That is, listeners may shift their attention to expected targets but do not necessarily exclude distractor objects in the scene from any internal set representation of likely continuations. Generating more or less specific expectations may therefore not require more or less effort, instead objects categorized as ’relevant’ (as opposed to ’probably not relevant’) may be assigned higher saliency within the external representation provided by the scene. How exactly such expectations for upcoming referents are represented, however, is subject to further research.

In sum, the visual context influences the expectancy for an object noun which, in turn, results in corresponding processing effort during retrieval. This shows that surprisal, and its associated processing effort, is not determined by the linguistic signal alone but rather reflects expectations derived on-line from the relevant visual environment in which the sentence is heard, capturing *situated* language processing. At the same time, we find no evidence for a “trade-off” theory of expectations derived from visual context in which the benefit of a more specific expectation (one that reduces referential entropy) would be paid earlier, when that expectation is generated. Instead, processing effort is invariant to the modulation of situatedly determined expectancy.

## Footnotes

1 Note that the N400 amplitude is considered here to index ’processing effort’ in broad terms, with no statements as to whether this effort is a by-product of facilitated word retrieval, word integration or other.

2 A pre-test assessed whether the pieces of clip art used in this experiment were indeed interpreted as related to ”their” verb. In an online web-form, 40 participants had to spontaneously decide whether or not an *object* was *“verb-able”*, by ticking a box stating “yes” or “no”. Images were used as experimental items if they were determined to be *“verb-able”* more than 90% of the time.

## References

[CR1] Altmann G, Kamide Y (1999). Incremental interpretation at verbs: Restricting the domain of subsequent reference. Cognition.

[CR2] Ankener, C., Sekicki, M., & Staudte, M. (2018). The influence of visual uncertainty on word surprisal and processing effort. *Frontiers in Psychology, 9*, 2387. 10.3389/fpsyg.2018.02387 PMC6302025 30618905

[CR3] Brouwer, H., Fitz, H., & Hoeks, J. (2012). Getting real about semantic illusions: rethinking the functional role of the p600 in language comprehension. *Brain Research, 1446*, 127–143. 10.1016/j.brainres.2012.01.055 22361114

[CR4] Dambacher M, Kliegl R (2007). Synchronizing timelines: Relations between fixation durations and N400 amplitudes during sentence reading. Brain research.

[CR5] Demberg V, Keller F (2008). Data from eye-tracking corpora as evidence for theories of syntactic processing complexity. Cognition.

[CR6] Demberg V, Sayeed A (2016). The frequency of rapid pupil dilations as a measure of linguistic processing difficulty. PLoS ONE.

[CR7] Federmeier KD, Wlotko EW, De Ochoa-Dewald E, Kutas M (2007). Multiple effects of sentential constraint on word processing. Brain Research.

[CR8] Frank S (2013). Uncertainty reduction as a measure of cognitive load in sentence comprehension. Topics in Cognitive Science.

[CR9] Frank, S. (2013b). Word surprisal predicts N400 amplitude during reading. In *Proceedings of the 51st annual meeting of the association for computational linguistics*.

[CR10] Frank, S., Otten, L., Galli, G., & Vigliocco, G (2015). The ERP response to the amount of information conveyed by words in sentences. *Brain and Language* (140), 1–11. 10.1016/j.bandl.2014.10.006 25461915

[CR11] Hale, J. (2001). A probabilistic Earley parser as a psycholinguistic model. In *Proceedings of the second meeting of the North American Chapter of the Association for Computational Linguistics on Language technologies* (pp. 1–8).

[CR12] Hale J (2016). Information-theoretical complexity metrics. Language and Linguistics Compass.

[CR13] Knoeferle P, Crocker MW, Pickering M, Scheepers C (2005). The influence of the immediate visual context on incremental thematic role-assignment: Evidence from eye-movements in depicted events. Cognition.

[CR14] Kutas M, Hillyard S (1980). Reading senseless sentences: Brain potentials reflect semantic incongruity. Science.

[CR15] Linzen T, Jaeger TF (2016). Uncertainty and expectation in sentence processing: Evidence from subcategorization distributions. Cognitive Science.

[CR16] Luck, S.J. (2014). *An introduction to the event-related potential technique*. The MIT Press.

[CR17] Maess B, Mamashli F, Obleser J, Helle L, Friederici AD (2016). Prediction signatures in the brain: Semantic pre-activation during language comprehension. Frontiers in Human Neuroscience.

[CR18] Marshall, S. P. (2000). Method and apparatus for eye tracking and monitoring pupil dilation to evaluate cognitive activity. US Patent, 6,090,051.

[CR19] Roark, B., Bachrach, A., Cardenas, C., & Pallier, C. (2009). Deriving lexical and syntactic expectation-based measures for psycholinguistic modeling via incremental top-down parsing. In *Proceedings of the 2009 conference on empirical methods in natural language processing* (vol. 1, pp. 324–333).

[CR20] Sekicki M, Staudte M (2018). Eye’ll help you out! How the gaze cue reduces the cognitive load required for reference processing. Cognitive Science.

[CR21] Shannon C (1949). Communication in the presence of noise. Proceedings of the IRE.

[CR22] Sharbrough F, Chatrian GE, Lesser RP, Lüders H, Nuwer M, Picton TW (1991). American Electroencephalographic Society guidelines for standard electrode position nomenclature. Journal of Clinical Neurophysiology.

[CR23] Smith N, Levy R (2013). The effect of word predictability on reading time is logarithmic. Cognition.

[CR24] Spivey, M. J., Richardson, D. C., & Fitneva, S. A. (2004). Thinking outside the brain: Spatial indices to visual and linguistic information. In J. M. Henderson, & F. Ferreira (Eds.) *The interface of language, vision, and action: Eye movements and the visual world* (pp. 161–189). New York: Psychology Press.

[CR25] Tourtouri, E., Delogu, F., & Crocker, M. W. (2015). ERP indices of situated reference in visual contexts. In *Proceedings of the 37th annual conference of the Cognitive Science Society* (pp. 2422–2427).

[CR26] Tourtouri, E., Delogu, F., Sikos, L., & Crocker, M. W. (2019). Rational over-specification in visually-situated comprehension and production. *Journal of Cultural Cognitive Science*, 1–28.

[CR27] Van Berkum JJ, Koornneef AW, Otten M, Nieuwland MS (2007). Establishing reference in language comprehension: An electrophysiological perspective. Brain Research.

[CR28] Wlotko EW, Federmeier KD (2012). So that’s what you meant! Event-related potentials reveal multiple aspects of context use during construction of message-level meaning. NeuroImage.

